# Supplementary material for: Intestinal fructose metabolism drives unsaturated fat absorption and synergizes with GLP-1 receptor agonism to promote weight loss
Source: bioRxiv. 2026 Jun 8:2026.06.03.729910. Preprint. [Version 1] doi: 10.64898/2026.06.03.729910 (PMC13278148; doi:10.64898/2026.06.03.729910)

bioRxiv preprint doi: https://doi.org/10.64898/2026.06.03.729910; this version posted June 8, 2026. The copyright holder for this preprint (which was not certified by peer review) is the author/funder, who has granted bioRxiv a license to display the preprint in perpetuity. It is made available under a CC-BY 4.0 International license.

Supplementary Figure 1. Dietary sucrose promotes adiposity without changing lean mass: A) Lean mass over time in KHK$^{+/+}$ animals exposed to different diet interventions over a period of 5 weeks. Low fat (black), high-fat high-sucrose (HFHS, red) or high-fat high-dextrose (HFHD, teal) diets (n= 10 mice per group). **B)** Linear regression analyses of average food intake (kcal) versus body weight over the final 48 hours in metabolic cages from the KHK$^{+/+}$ mice (n= 4 mice per group). **C)** Lean mass over time in KHK$^{-/-}$ mice exposed to different diet interventions over a period of 5 weeks (n= 9 mice per group), *(HFHS vs HFHD), *p=0.0264, ** p= 0.0045. **D)** Linear regression analyses of average food intake (kcal) versus body weight over the final 48 hours in metabolic cages from the KHK$^{-/-}$ mice (n= 4 mice per group). **E)** After five weeks of different diet exposure, euthanasia was performed, and tissues such as gonadal white adipose tissue (gWAT), and **F)** Gastrocnemius (gastroc), were collected and weighed (n= 9-10 mice per group). Bar graphs showing cumulative **H)**^{13}C-glucose and **G)** ^{13}C-fructose oxidation efficiency (nmol/mg) in KHK^{+}/$^{+}$ and KHK^{-}/$^{-}$ animals treated with vehicle (methylcellulose, purple) or a ketohexokinase inhibitor (KHKi, blue) (n= 6-8 mice per group). Data are presented as mean ± SEM; individual data points are overlaid. *P* values were calculated using one-way analysis of variance (ANOVA) with Dunnett's multiple comparison test or two-way ANOVA with Tukey's multiple comparison test.

Supplementary Figure 2. Dietary sucrose promotes weight gain in C57BL/6J female and can be prevented by KHK deletion. Female KHK$^{+/+}$ and KHK$^{-/-}$ animals received a low fat (black), high-fat high-sucrose (HFHS, red) or high-fat high-dextrose

bioRxiv preprint doi: https://doi.org/10.64898/2026.06.03.729910; this version posted June 8, 2026. The copyright holder for this preprint (which was not certified by peer review) is the author/funder, who has granted bioRxiv a license to display the preprint in perpetuity. It is made available under a CC-BY 4.0 International license.

(HFHD, teal) diets for 5 weeks. **A)** Female $KHK^{+/+}$ and **B)** Female $KHK^{-/-}$ body mass over time. **C)** Female $KHK^{+/+}$ and **D)** Female $KHK^{-/-}$ lean mass over time. **E)** Female $KHK^{+/+}$ and **F)** Female $KHK^{-/-}$ fat mass overt time. After five weeks of different diet exposure, euthanasia was performed, and tissues such as **G)** Gonadal white adipose tissue (gWAT) and **H)** Gastrocnemius (gastroc) were collected and weighed. Data are presented as mean ± SEM; individual data points are overlaid. *P* values were calculated using two-way analysis of variance (ANOVA) with Tukey's multiple comparison test or one-way with Dunnett's multiple comparison test. * p=0.0336. *(Low Fat vs HFHS). n=5 mice per group.

Supplementary Figure 3. Small molecule PF-06835919 led to no changes in body weight in $KHK^{-/-}$ mice under a HFHS diet: A) Inguinal white adipose tissue (iWAT), **B)** Gonadal white adipose tissue (gWAT) and **C)** Liver from obese mice after a month of oral treatment of PF-06835919 were collected at the end of the experiment (n=7 mice per group). **D)** Cumulative food intake (g) over a 48-hour period shows decreased energy consumption in KHKi group, when compared to HFHS (*) or to HFHD ($^{\#}$) group. **E)** Total energy expenditure (kcal/h) measured over 48 hours by indirect calorimetry reveals no significant differences among groups, with preserved circadian rhythmicity. **F)** KHKi treatment decreases caloric absorption (Kcal) compared to HFHS or HFHD groups (n=3 mice per group). **G)** ^{3}H-Oleic acid levels were measured at the end of the experiment in different tissue sections such as liver, proximal intestine (small intestine = SI), distal intestine and colon in the different experimental groups (n=2-4 mice per group). No off-target effects were observed in **H)** Body weight, **I)** Fat mass and **J)** Lean

bioRxiv preprint doi: https://doi.org/10.64898/2026.06.03.729910; this version posted June 8, 2026. The copyright holder for this preprint (which was not certified by peer review) is the author/funder, who has granted bioRxiv a license to display the preprint in perpetuity. It is made available under a CC-BY 4.0 International license.

mass over a period over a period of 4 weeks (n=8-9 mice per group) when KHK$^{-/-}$ mice were treated with a HFHS diet + PF-06835919. HFHS: High-fat high-sucrose diet + vehicle, HFHD: high-fat high-dextrose diet + vehicle, HFHS + KHKi: High-fat high sucrose diet + PF-06835919. Volcano plots showing differentially abundant lipid species in **K)** feces (n=5 mice per group) and **L)** serum (n=3 control, n=4 HFHS + KHKi) of DIO mice fed a HFHS diet for 15 weeks. Each dot represents one lipid species, colored by degree of unsaturation. DB: double bonds, MUFA: monounsaturated, PUFA: polyunsaturated. The x-axis shows the $\log_2$ fold change (KHKi/Control) and the y-axis shows $-\log_{10}$ (*p*-value). **M)** Convergent serum TG species depleted by KHKi and elevated by HFCS (High fructose corn syrup). Mirror bar chart showing $\log_2$ fold changes for 12 triglyceride species (1-7 double bonds) that are significantly altered in opposite directions by KHK inhibition (blue, left; KHKi *vs*. control, n=3/4) and HFCS feeding (orange, right; HFCS *vs*. control, from Fowle-Grider *et al*[34]., n=4/6). Species are ordered by the magnitude of KHKi depletion. Data are presented as mean ± SEM; individual data points are overlaid. *P* values were calculated using one-way analysis of variance (ANOVA) with Dunnett's multiple comparison test or two-way ANOVA with Tukey's multiple comparison test. *: *p*< 0.05, **/##: *p*<0.001, ***: *p*<0.001.

Supplementary Figure 4. Villus zone-specific expression of functionally related fructolysis and lipid uptake/metabolism gene groups in mouse and human small intestine: A) Mouse and **B)** Human enterocyte expression of genes related to fructolysis and chylomicron assembly along the proximal-distal intestine axis. Value represents the mean scaled expression of each gene, colored by domain, with

bioRxiv preprint doi: https://doi.org/10.64898/2026.06.03.729910; this version posted June 8, 2026. The copyright holder for this preprint (which was not certified by peer review) is the author/funder, who has granted bioRxiv a license to display the preprint in perpetuity. It is made available under a CC-BY 4.0 International license.

surrounding shade standard error bounds, across intestinal segments. Segment positions are numbered (x axis), and the positions of domain boundaries are noted with shade lines. Original data from Zwick *et al*.[9] **C)** Normalized expression of genes involved in four lipid-handling functions across seven villus zones (crypt to tip) in mouse small intestinal enterocytes, replotted from Moor *et al.,*[35]. Chylomicron assembly genes (*Mttp*, *Apob*, *Dgat2*, *Cd36*; blue) and lipid droplet/fatty acid uptake genes (*Plin2, Fabp2, Mogat2, Dgat1, Cideb*; green) are enriched toward the villus tip. Fructolysis genes (*Khk, Slc2a5, Aldob*; red) peak at mid-villus zones (v2-v3). Apolipoproteins (*Apoa4, Apoa1;* purple) show a progressive tip-biased gradient. **D)** Normalized expression of orthologous human gene groups across six villus zones (crypt to tip) in the proximal small intestine, replotted from Harnik *et al*.,[8] . Chylomicron assembly genes (*MTTP, APOB, DGAT2, CD36;* blue) are tip-based, fructose metabolism genes (*KHK, ALDOB;* red) peak at mid-villus zone (V3-V5) and lipid droplet assembly genes (*BSCL2, TMEM159;* green) are crypt-biased.

Supplementary Figure 5. Semaglutide alone or in combination with PF-06835919 enhances fat and lean mass weight loss under a HFHS diet: At the end of the experiment, necropsy was performed and tissues such as **A)** iWAT (inguinal white adipose tissue), **B)** gWAT (gonadal white adipose tissue), **C)** Liver, **D)** Gastroc (Gastrocnemius), **E**) Soleus and **F)** Kidney were collected and weighted (n=9-10 mice per group)**. G)** Linear regression analyses of TEE (kcal) versus body weight (g) and **H)** Cumulative food intake (g) over the final 48 hours in metabolic cages (n=8 mice per group)**.** Data are presented as mean ± SEM; individual data points are overlaid. *P*

bioRxiv preprint doi: https://doi.org/10.64898/2026.06.03.729910; this version posted June 8, 2026. The copyright holder for this preprint (which was not certified by peer review) is the author/funder, who has granted bioRxiv a license to display the preprint in perpetuity. It is made available under a CC-BY 4.0 International license.

values were calculated using one-way analysis of variance (ANOVA) with Dunnett's multiple comparison test. $^{\#/*}$p=0.005, $^{\#}$HFHS vs. semaglutide + KHKi, *HFHS vs. semaglutide

Supplementary Figure 6. Small molecule PF-06835919 delays fat mass regains after semaglutide withdrawal: C57BL/6J male mice were fed a high-fat high-sucrose (HFHS; black) diet for 15 weeks to develop a DIO model. After this time, mice were randomized and subdivided in three different groups: continued HFHS (vehicle), HFHS + Semaglutide (30 nmol/kg; purple), or HFHS + Semaglutide + KHKi (PF-06835919; 50 mg/kg; green). **A)** Fat mass (g) and **B)** Lean mass (g) were measured longitudinally by body composition analysis. Tissue collection such as **C)** Inguinal white adipose tissue (iWAT), **D)** Gonadal white adipose tissue (gWAT), **E)** Liver, **F)** Gastrocnemius (gastroc), **G)** Soleus and **H)** Kidney from DIO mice after the withdrawal phase were collected at the end of the experiment (n=5-7 mice per group). Data are presented as mean ± SEM; individual data points are overlaid. *P* values were calculated using one-way analysis of variance (ANOVA) with Dunnett's multiple comparison test. $^{\#}p=0.0215$, $^{\#}$HFHS vs Semaglutide+ KHKi.

Supplementary Table 1: Effects of HFHS feeding diet on female C57BL/6J and KHK$^{-/-}$ mice

	Female C57BL/6J			Female KHK$^{-/-}$		
	Low Fat	HFHS	HFHD	Low Fat	HFHS	HFHD
Insulin ng/mL	0.98 ± 0.27	1.01 ± 0.25	1.19 ± 0.51	1.47 ± 0.50	0.49 ± 0.10	1.72 ± 0.59
Leptin pg/mL	1350.70 ± 485.64	5775.88 ± 2611.70	1686.46 ± 480.45	3064.44 ± 558.98	3942.42 ± 858.41	1870.06 ± 472.90
Glucose (mg/dL)	186.2 ± 7.86	252.2 ± 14.14**	179.4 ± 5.89~~~	155.2 ± 8.40	134.2 ± 6.18	149.2 ± 11.27
Triglycerides (mg/dL)	46.28 ±3.62	53.28 ± 4.36	48.07 ±4.59	41.17±6.28	48.76 ± 6.01	29.46 ± 2.35
Cholesterol (mg/dL)	56.43 ± 3.87	65.79 ± 9.30	79.63 ± 7.70	48.22 ± 2.19	57.56 ± 1.54	50.32 ± 7.13
NEFA (mEQ/L)	0.41 ± 0.06	0.52 ± 0.08	0.44 ± 0.06	0.30 ± 0.06	0.46 ± 0.08	0.38 ± 0.09

**0.0014, ~~~0.0006. *Low Fat vs HFHS, ~HFHS vs HFHD. Ordinary one-way ANOVA with Tukey's multiple comparisons test. Data are mean ± SEM. n= 4-5 mice per group

bioRxiv preprint doi: https://doi.org/10.64898/2026.06.03.729910; this version posted June 8, 2026. The copyright holder for this preprint (which was not certified by peer review) is the author/funder, who has granted bioRxiv a license to display the preprint in perpetuity. It is made available under a CC-BY 4.0 International license.

Echeverria & Ahmed et al. Supplementary 1

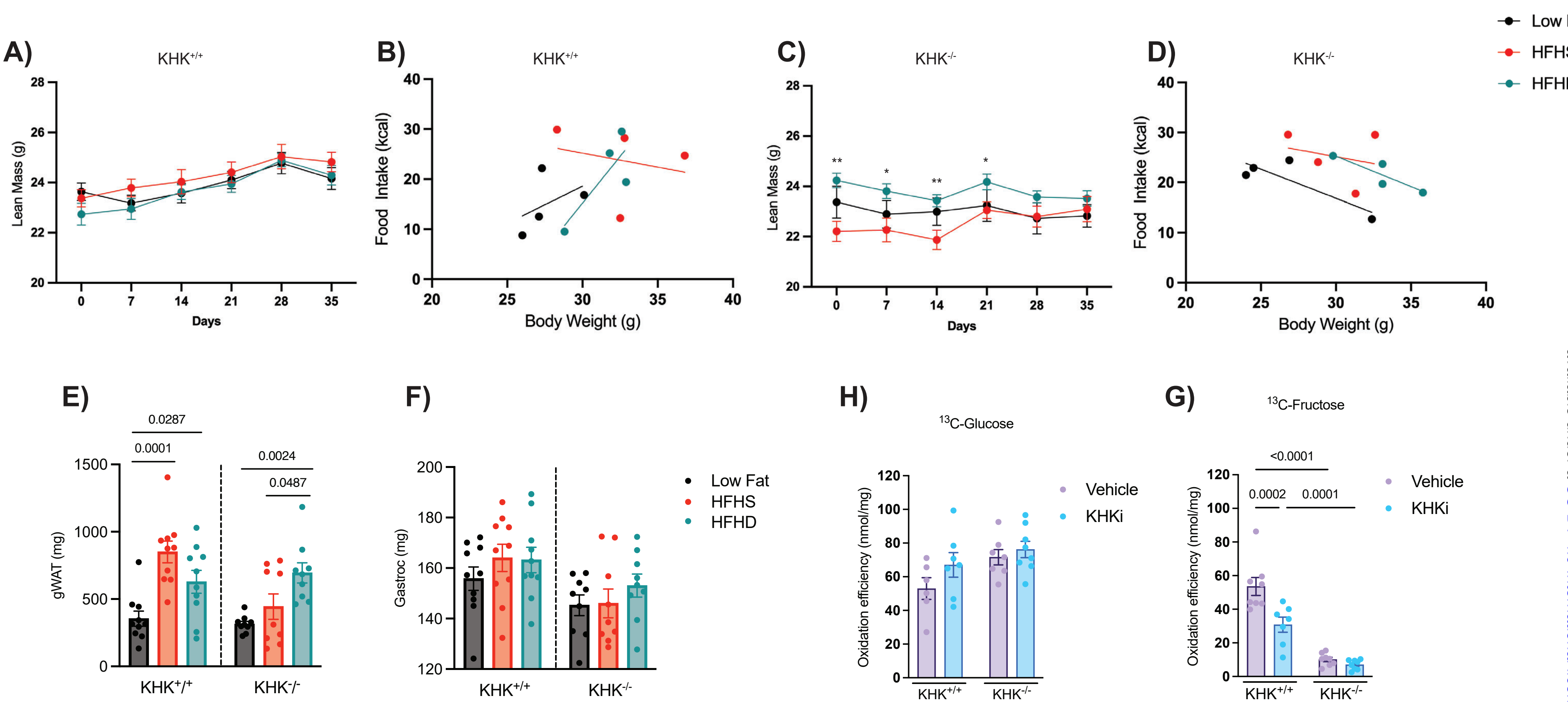

bioRxiv preprint doi: https://doi.org/10.64898/2026.06.03.729910; this version posted June 8, 2026. The copyright holder for this preprint (which was not certified by peer review) is the author/funder, who has granted bioRxiv a license to display the preprint in perpetuity. It is made available under a CC-BY 4.0 International license.

bioRxiv preprint doi: https://doi.org/10.64898/2026.06.03.729910; this version posted June 8, 2026. The copyright holder for this preprint (which was not certified by peer review) is the author/funder, who has granted bioRxiv a license to display the preprint in perpetuity. It is made available under a CC-BY 4.0 International license.

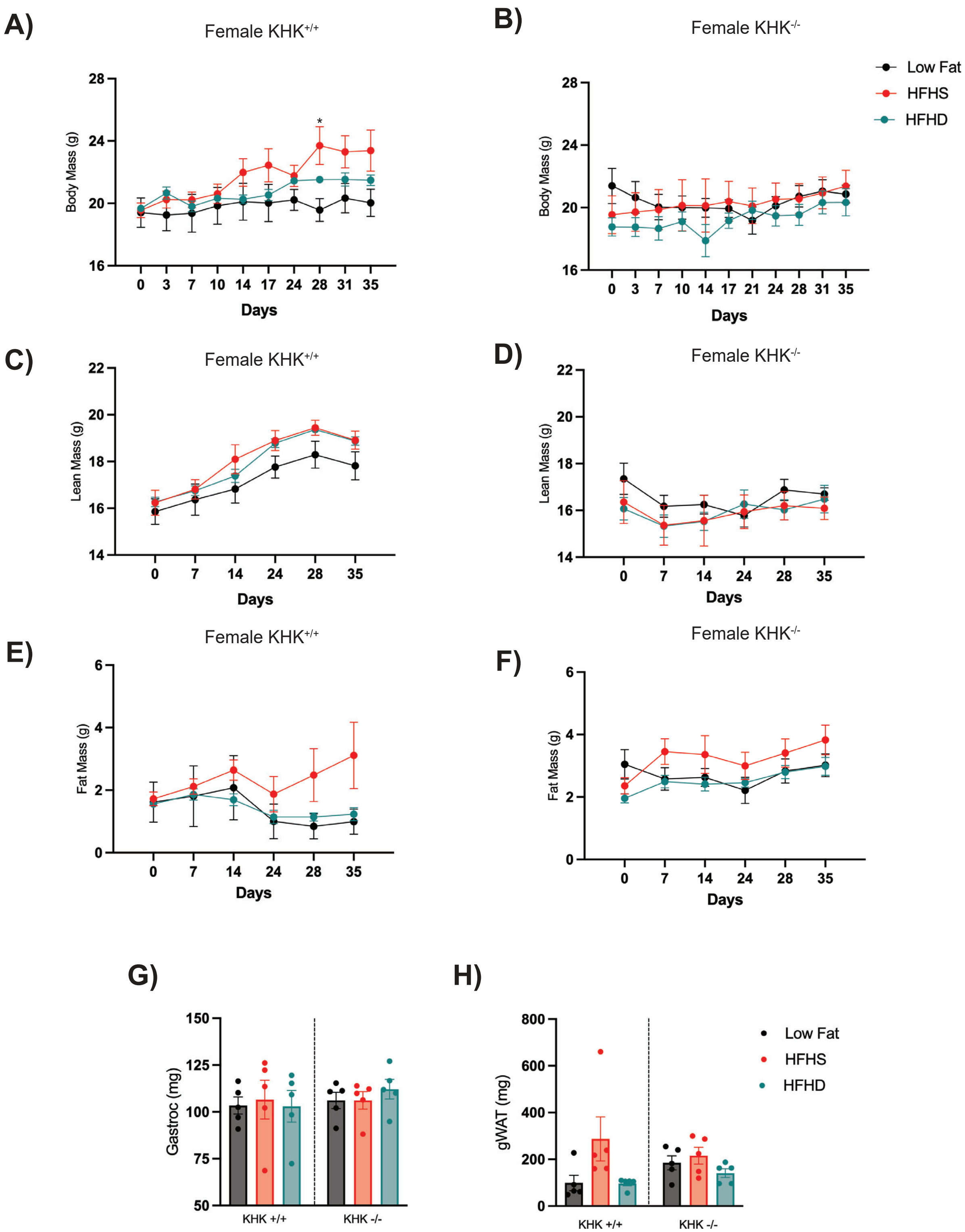

Echeverria & Ahmed et al. Supplementary 3

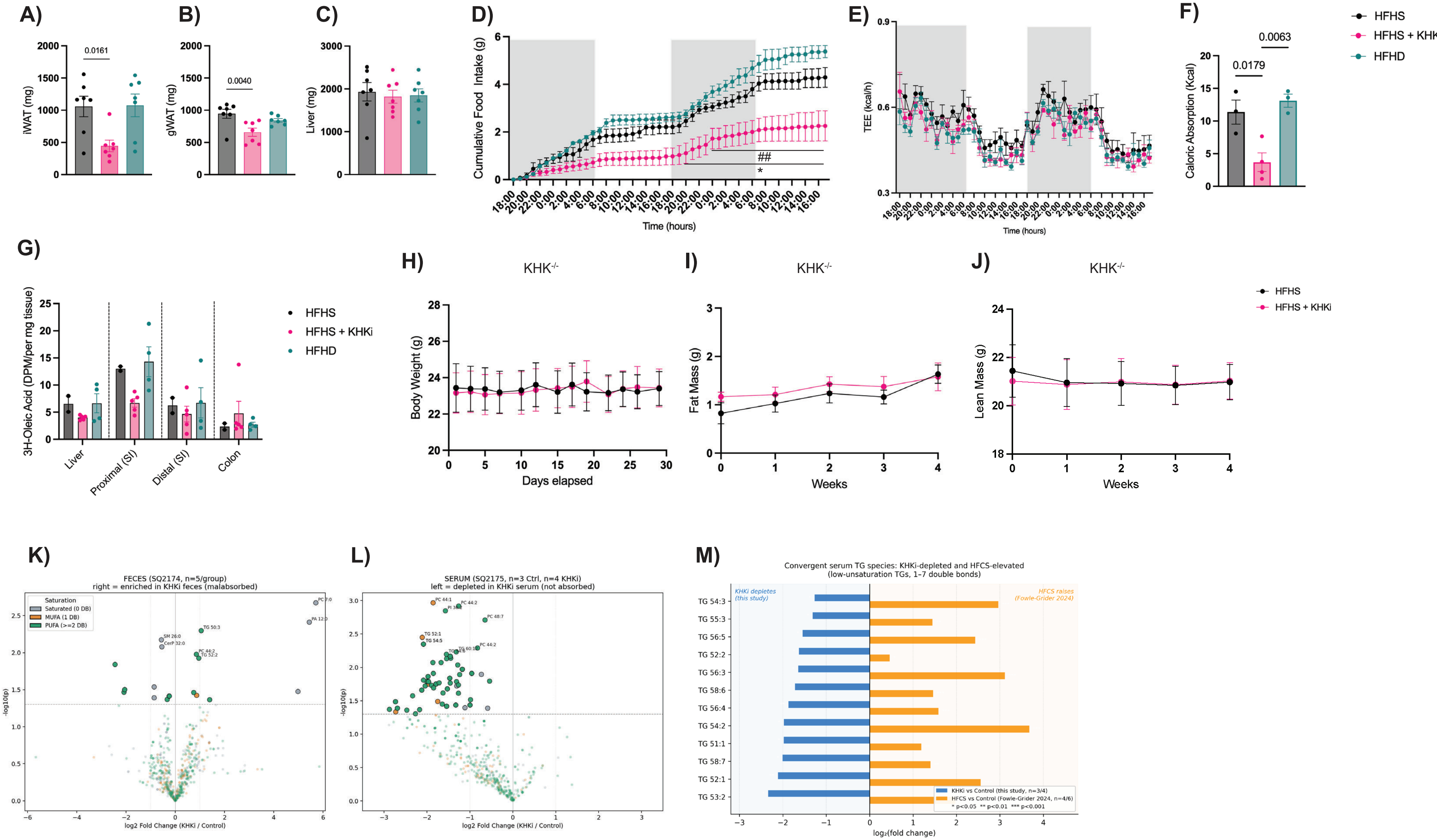

bioRxiv preprint doi: https://doi.org/10.64898/2026.06.03.729910; this version posted June 8, 2026. The copyright holder for this preprint (which was not certified by peer review) is the author/funder, who has granted bioRxiv a license to display the preprint in perpetuity. It is made available under a CC-BY 4.0 International license.

bioRxiv preprint doi: https://doi.org/10.64898/2026.06.08.729910; this version posted June 8, 2026. The copyright holder for this preprint (which was not certified by peer review) is the author/funder, who has granted bioRxiv a license to display the preprint in perpetuity. It is made available under a CC-BY 4.0 International license.

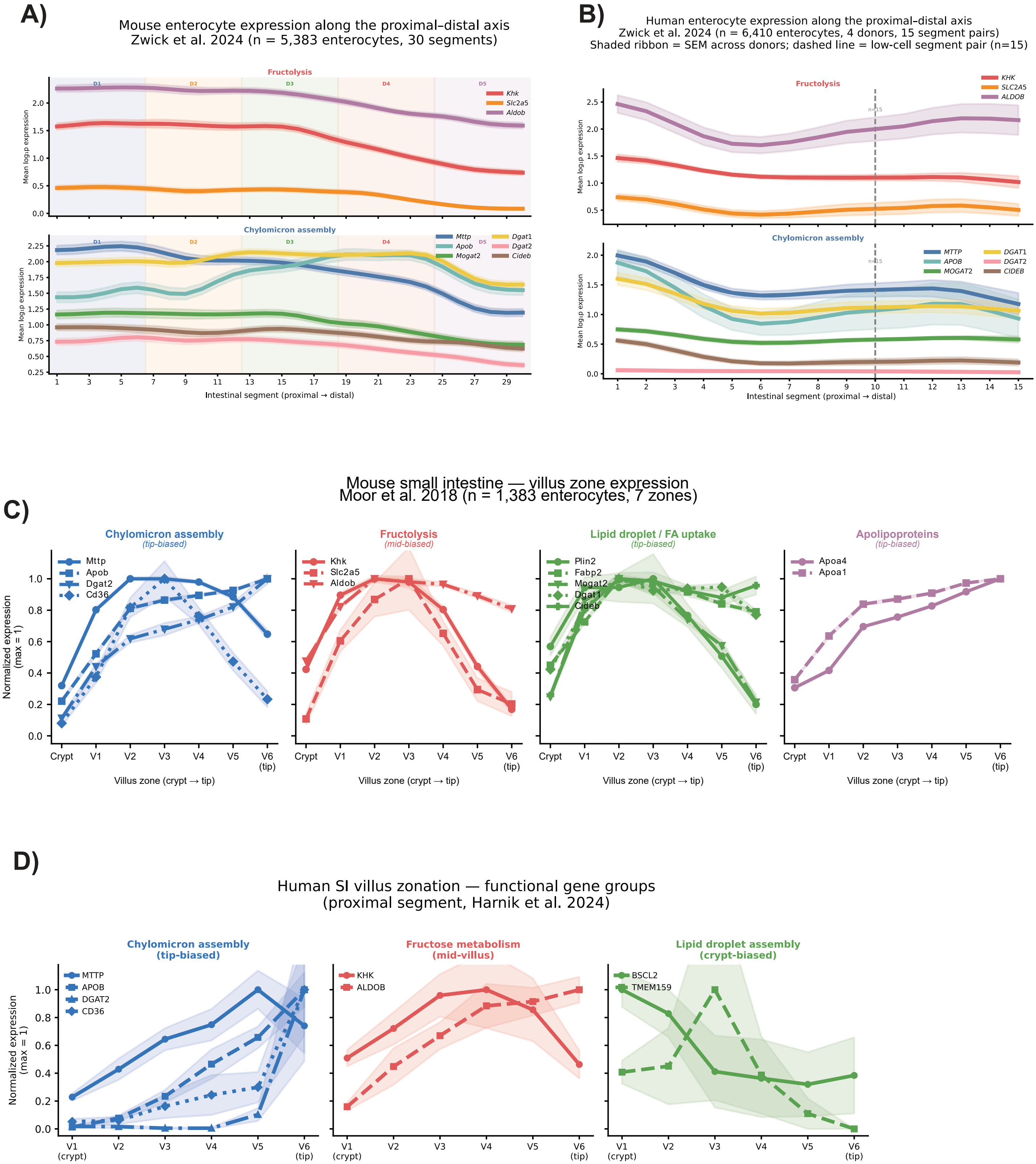

bioRxiv preprint doi: https://doi.org/10.64898/2026.06.08.729910; this version posted June 8, 2026. The copyright holder for this preprint (which was not certified by peer review) is the author/funder, who has granted bioRxiv a license to display the preprint in perpetuity. It is made available under a CC-BY 4.0 International license.

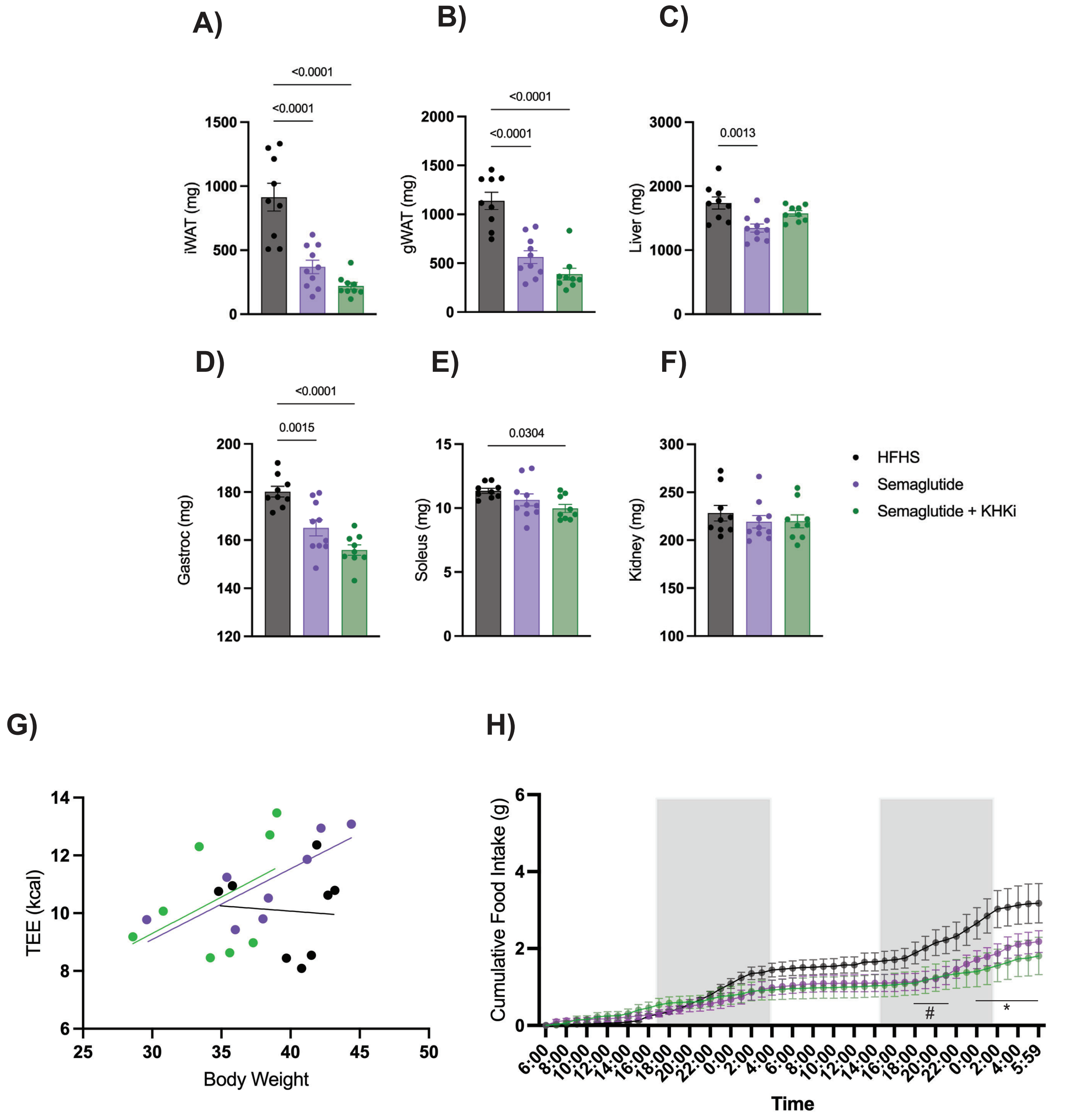

bioRxiv preprint doi: https://doi.org/10.64898/2026.06.03.729910; this version posted June 8, 2026. The copyright holder for this preprint (which was not certified by peer review) is the author/funder, who has granted bioRxiv a license to display the preprint in perpetuity. It is made available under a CC-BY 4.0 International license.

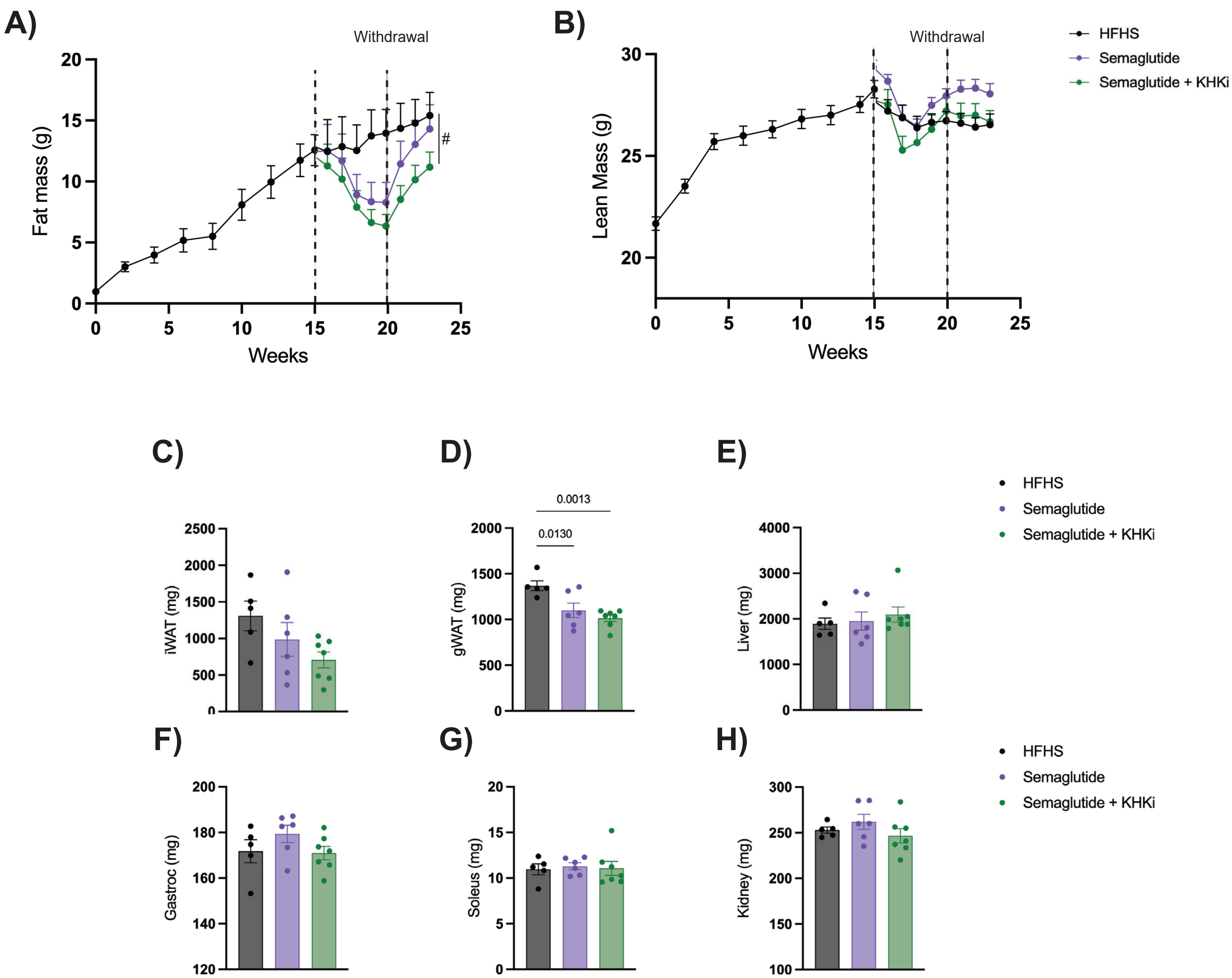

Supplement: Supplementary file 1 [file NIHPP2026.06.03.729910v1-supplement-1.pdf]
